# Journal Club Article: The STEROHCA trial – Optimizing post resuscitation haemodynamics by prehospital high dose corticosteroids

**DOI:** 10.1016/j.resplu.2025.101078

**Published:** 2025-08-28

**Authors:** Sebastian Billig, Jennifer Dermer, Lykke Kjærsgaard, Joseph Hamilton, Joyce Yeung

**Affiliations:** a Department of Anesthesiology, RWTH Aachen University Hospital, Aachen, Germany; b School of Health-Discipline of Nursing, University of the Sunshine Coast, Australia; c Department of Anaesthesiology and Intensive Care, Aarhus University Hospital, Aarhus, Denmark; d Department of Community, Social Justice and Health, University of Worcester, Worcester, United Kingdom; e Warwick Medical School, University of Warwick, United Kingdom; f Department of Critical Care, University Hospitals Birmingham NHS Foundation Trust, United Kingdom

**Figure:**
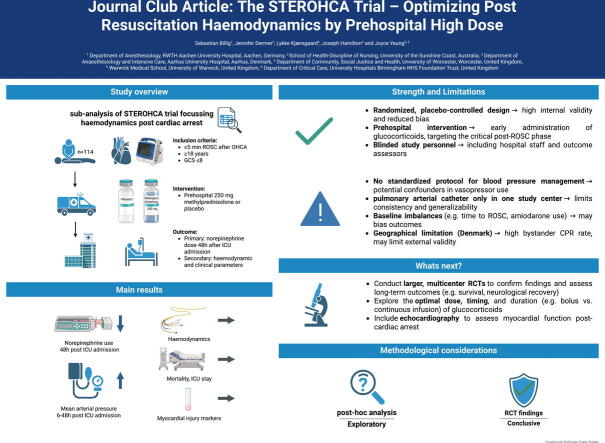

## Which knowledge gap does this study try to fill out?

Despite significant advances in post-cardiac arrest (CA) care, most patients who achieve a return of spontaneous circulation (ROSC) after CA experience major haemodynamic instability.^1^, ^2^ This instability refers to a complex of pathophysiological changes during the post cardiac arrest syndrome and is caused by a systemic inflammation and myocardial dysfunction.^1^, ^2^, ^3^ Furthermore, a low arterial blood pressure post-ROSC is associated with reduced survival.^4^ However, excessive use of vasopressors, which are typically used to maintain an adequate perfusion pressure, can lead to adverse outcomes.^5^ Glucocorticoids have well-known anti-inflammatory properties and have been shown to reduce vasopressor requirements in septic shock patients.^6^ Until now their role in post CA care remains unclear.^7^, ^8^ The study ‘Effects of prehospital high-dose glucocorticoid on hemodynamics in patients resuscitated from out-of-hospital cardiac arrest: a sub-study of the STEROHCA trial’ by *Obling et al*., published in *Critical Care*, investigated whether prehospital administration of high-dose glucocorticoids improves haemodynamics and reduces vasopressor amounts in comatose patients resuscitated from out-of-hospital cardiac arrest (OHCA).^9^

## What was the design of the study?

This study was a post-hoc analysis of the STEROHCA trial, an investigator-initiated, randomized, placebo-controlled, multicenter trial conducted in Denmark from 2020 to 2022.^10^ The main STEROHCA trial analyzed neuron-specific enolase and interleukin-6 levels in 137 OHCA survivors, who were randomized to receive either a single prehospital dose of 250 mg methylprednisolone or placebo after ROSC. Adults aged 18 years and older were eligible if they were resuscitated from OHCA due to a presumed cardiac cause, were unconscious (GCS ≤ 8), and had a ROSC for at least 5 min. Blinding was maintained across all involved personnel, including hospital staff, outcome assessors, patients, and family members.

This sub-analysis included 114 of the 137 patients from the main trial. The primary outcome was the cumulative norepinephrine use from intensive care unit (ICU) admission to 48 h post-admission. Secondary outcomes included mean arterial blood pressure, heart rate, vasoactive-inotropic score, haemodynamic parameters of pulmonary artery catheter measurements, and other clinical parameters.

## What were the key findings of the study?

Patients in the glucocorticoid group exhibited a significantly lower cumulative norepinephrine requirement over the first 48 h in the ICU, along with a higher mean arterial pressure 12–24 h post-ICU admission, and a lower mean arterial pressure/ vasoactive-inotropic score ratio. There were no significant differences in the pulmonary artery catheter-derived measurements. Furthermore, no differences were seen in ICU treatment durations, ventilator days, mortality rates or serum parameters of myocardial injury (Troponin T and I, Creatine Kinase MB and NT-proBNP). In summary, the study found that although prehospital administration of high-dose glucocorticoids mitigates the need for vasopressors during the immediate post-resuscitation period, it did not yield a definitive advantage in terms of mortality or other clinical outcomes evaluated within this patient cohort.

## Are there any important methodological considerations to learn for the study?

As the authors state, the statistical analyses of this sub-analysis of the STERHOCA trial were primarily post-hoc and not part of the original study protocol.^11^ This implies that the results of the hemodynamic analyses in this sub-analysis are not sufficient to conclusively answer questions regarding the hemodynamic effects of prehospital corticosteroids administration after cardiac arrest.

It is important to make a clear distinction between predefined (prespecified) and exploratory (post-hoc) analyses in clinical research.^12^ Prespecified analyses are those defined in the study protocol before data collection begins, usually based on an initial hypothesis. While some prespecified analyses may be supported by formal sample size calculations, many are not. Post-hoc analyses are typically developed after seeing the data or during the conduct of the trial, often based on emerging observations or insights. While post-hoc analyses can provide valuable insights such as identifying trends, raising new hypotheses, or exploring subgroups, they carry a risk of false-positive or false-negative findings due to multiple testing and data-driven selection.^12^ Therefore, they should always be interpreted cautiously. Transparent labeling of post-hoc analyses is essential to maintain scientific integrity and avoid overinterpretation of results.

Both prespecified and post-hoc secondary analyses should be considered hypothesis-generating in nature. Confirmatory conclusions and causal statements should only be based on the primary endpoints of a randomized controlled trial for which the study is adequately powered.

## What are the most important strengths and limitations of the study?

This study provides valuable insights into the early hemodynamic effects of glucocorticoids in OHCA patients and is notable for its well-conducted, randomized, placebo-controlled design. A major strength is the immediate administration of the intervention in the prehospital setting, reducing delays in therapy initiation. The randomized controlled design enhances the internal validity of the study and minimizes bias.^13^ Furthermore, the inclusion of repeated hemodynamic measurements via pulmonary arterial catheters allows for high-quality, operator-independent data collection using a gold-standard reference method.^14^

However, certain limitations must be acknowledged. This study lacked a predefined treatment protocol for blood pressure management, which is particularly relevant given that the primary outcome was vasopressor requirement. Since the original trial was a placebo-controlled trial, the risk of differential bias or confounding between groups is inherently minimized by the study design. Additionally, pulmonary artery catheter measurements were only conducted in one of the two study centres. Patients who were equipped with a pulmonary artery catheter were younger and more often received invasive myocardial revascularisation (*p* < 0.001), potentially limiting the consistency of haemodynamic data. The study’s geographic limitation, within a region with a high prevalence of bystander cardiopulmonary resuscitation, restricts external validity and its generalizability. Moreover, baseline imbalances between the groups such as longer time to ROSC and higher prehospital amiodarone use in the glucocorticoid group could have influenced the results.

## How will the results affect your clinical practice?

The findings of the study by *Obling et al*. suggest that early administration of high-dose glucocorticoids can reduce vasopressor requirements and improve hemodynamic stability in post-CA patients. However, this did not translate into a significant mortality benefit. While these findings may prompt reconsideration of current protocols to include glucocorticoid therapy, caution is warranted due to the study limitations. Before implementing glucocorticoid therapy into clinical practice, it would be prudent to wait for larger trials confirming these results. Furthermore, potential adverse effects of glucocorticoids e.g. infection risk or metabolic dysregulation, must be considered.^15^ Notably, *Obling et al.* reported significantly higher metabolic adverse effects in the methylprednisolone group (45 % vs 12 %, *p* < 0.001). In the meantime, clinicians should continue to optimize hemodynamic management in OHCA patients and consider the role of inflammation in post-resuscitation care.

## What do you see as the next steps in research?

Future research should focus on larger, multicenter randomized controlled trials to validate these findings and assess long-term clinical outcomes, including survival and neurological function. Echocardiography should be incorporated into study protocols to assess the effects of steroids on myocardial function after OHCA.^16^ Additionally, research should explore optimal dosing and timing strategies, including whether continuous glucocorticoid therapy offers superior benefits compared to a single-dose approach. Investigating biomarkers for patient selection and targeted therapies may further refine the role of glucocorticoids in post-CA management.

## Declaration of generative AI in scientific writing

During the preparation of this work the authors used DEEP L to improve readability and language of the manuscript. After using this tool, the authors reviewed and edited the content as needed and take full responsibility for the content of the published article.

## CRediT authorship contribution statement

**Sebastian Billig:** Writing – review & editing, Writing – original draft, Visualization, Project administration, Methodology, Conceptualization. **Jennifer Dermer:** Writing – review & editing, Writing – original draft, Visualization, Methodology, Conceptualization. **Lykke Kjærsgaard:** Writing – review & editing, Writing – original draft, Visualization, Methodology, Conceptualization. **Joseph Hamilton:** Writing – review & editing, Writing – original draft, Visualization, Methodology. **Joyce Yeung:** Writing – review & editing, Writing – original draft, Supervision, Methodology, Conceptualization.

## Funding

This research did not receive any specific grant from funding agencies in the public, commercial, or not-for-profit sectors.

## Declaration of competing interest

The authors declare the following financial interests/personal relationships which may be considered as potential competing interests: “Joyce Yeung: ILCOR Honorary Secretary Unpaid, ILCOR scientific advisory committee member Unpaid, ERC SEC ALS member Unpaid, Editorial Board Member. Sebastian Billig: Abiomed Europe GmbH Research Grant, Clinician Scientist Program RWTH Aachen, The other authors declare no competing interests”.
